# Numerical modeling of high-intensity focused ultrasound-mediated intraperitoneal delivery of thermosensitive liposomal doxorubicin for cancer chemotherapy

**DOI:** 10.1080/10717544.2019.1660435

**Published:** 2019-09-16

**Authors:** Mohsen Rezaeian, Amir Sedaghatkish, M. Soltani

**Affiliations:** a Department of Mechanical Engineering, K. N. Toosi University of Technology, Tehran, Iran;; b Department of Mechanical Engineering, Isfahan University of Technology, Isfahan, Iran;; c Advanced Bioengineering Initiative Center, Computational Medicine Center, K. N. Toosi University of Technology, Tehran, Iran;; d Department of Electrical and Computer Engineering, University of Waterloo, Waterloo, Canada;; e Centre for Biotechnology and Bioengineering (CBB), University of Waterloo, Waterloo, Canada;; f Cancer Biology Research Center, Cancer Institute of Iran, Tehran University of Medical Sciences, Tehran, Iran

**Keywords:** Thermosensitive liposomes, intraperitoneal (IP) chemotherapy, high-intensity focused ultrasound (HIFU), microvascular permeability, solid tumor, drug delivery

## Abstract

Although intraperitoneal chemotherapy (IPC) has been suggested as a promising method for the management of peritoneal dissemination (PD) of ovarian or colorectal cancers, the actual clinical use of this method has been restricted due to such problems as poor drug penetration into the tumor and high side effects. It is, therefore, necessary to develop new strategies to improve the efficacy of this approach. In the present work, a new strategy is proposed based on intraperitoneal (IP) injection of thermosensitive liposomal doxorubicin (TSL-Dox) with triggered release by mild hyperthermia induced by high intensity focused ultrasound (HIFU). A computational model is developed to evaluate the proposed drug delivery system. Results show an order of magnitude increase in drug penetration depth into the tumor compared to the conventional IP delivery. Furthermore, the effects of thermal conditions applied to the tumor, TSL size, tumor vessel permeability, and tumor size are investigated. Results indicate an improved efficiency of the drug delivery by expanding the heated region, yet, it increases the risk of unintentional TSL drug load release in the peritoneal cavity. Results also indicate that smaller TSLs have better treatment outcome. However, there is a significant reduction in treatment efficacy for TSLs with sizes smaller than the vessel wall pore size. Thus, tuning the size of TSL should be based on the tumor microvascular permeability. The simulation results suggest that the TSL-Dox delivery system in smaller tumors is far advantageous than larger ones. Results of our model can be used as guidelines for future preclinical studies.

## 1. Introduction

Peritoneal dissemination (PD) is one of the most serious consequences of patients with peritoneal carcinomatosis (PC). The predicted quality of life in these types of cancers is very poor and the five-year survival rate is less than 40% for advanced ovarian cancer and less than 12.5% for colorectal cancers (Burges & Schmalfeldt, 2011; Favoriti et al., 2016). Management approaches of PD have been associated with many changes over the past three decades. In the 1980s, chemotherapy by systematic injection with a palliative approach was associated with predicting an expected survival of less than a few months. However, using newer methods, such as cytoreductive surgery (CRS), along with hyperthermic intraperitoneal chemotherapy (HIPEC) for selected patients has provided a long-term survival rates, which can even lead to complete treatment in some cases (Sadeghi et al., 2000; Montori et al., 2014; Sloothaak et al., 2014; Wright et al., 2015). This combined treatment method has become a standard of care only for colorectal peritoneal metastasis under certain conditions with limited spread of the disease (Mohamed et al., 2011; Bhatt, 2018). In addition, peritoneal recurrence is common even after complete implementation of CRS and HIPEC (Bijelic et al., 2007; Königsrainer et al., 2013), which is over 50% for patients with pseudomyxoma peritonei (PMP), ovarian cancer, and mesothelioma. The recurrence after CRS and HIPEC is considered as a failure for treatment (van Oudheusden et al., 2015). Therefore, there is generally a need for a therapeutic strategy for effective PD management, particularly for patients who are not candidates for combined CRS and HIPEC treatment.

One of the most important considerations is to improve the intraperitoneal chemotherapy (IPC) efficiency. IPC transfers high amounts of anticancer drugs to peritoneal site, thereby, directly exposing peritoneal neoplasms to high concentrations of these drugs (Lambert, 2015), unlike intravenous (IV) injection in which the drug is delivered by translocation through the bloodstream. IPC is completed within 30–120 minutes, which is considered a short time for injection (De Smet et al., 2013) causing insufficient drug delivery to the tumor. In addition, low molecular weight drugs are rapidly absorbed by capillaries and enter the circulatory system (Hirano et al., 1985). Although a drug entered the circulation may have little secondary therapeutic effects, the systemic effect of the drug should be low enough to minimize its side effects (De Smet et al., 2013). In addition, the tumor-specific pathophysiology including the denseness of extracellular matrix (ECM), lack of an effective lymphatic system, and a leaky and spatially heterogeneous microvasculature lead to a high interstitial pressure in the tumor, followed by an outward convective flux in the tumor periphery (Shamsi et al., 2018), all of which inhibit the effective drug penetration to the tumor interior. A low drug penetration depth in the tumor is one of the main weaknesses of IPC. In order to increase the treatment efficacy, the drug delivery system used for IPC should consider all the above-mentioned restrictions, including limitations related to side effects and poor drug penetration.

Liposomes are drug carriers used to improve drug delivery and reduce the side effects of chemotherapy by releasing their load in a pre-designed, controllable manner (Zhan & Wang, 2018). Thermosensitive liposomes (TSLs) in combination with mild local hyperthermia (HT) have been shown to enhance the systemic chemotherapy(Willerding et al., 2016; Lokerse et al., 2018). TSL is a drug carrier that releases its content at a threshold temperature of about 40 °C (Kong et al., 2000; Needham & Dewhirst, 2001; Li et al., 2010). The drug release rate in this system is strongly dependent on the local temperature of the tissue. Localized HT can be created by using HIFU as a controllable, noninvasive, and high-precision method (ter Haar & Coussios, 2007; Staruch et al., 2011; Tempany et al., 2011). The use of TSL triggered by HIFU-induced mild HT to reduce side toxicity and improve drug delivery in IV chemotherapy has been widely considered in preclinical studies (Ponce et al., 2006; Dromi et al., 2007; Staruch et al., 2012; Hijnen et al., 2014; Centelles et al., 2018). Although no report is available on such a drug delivery system in IP injection, employing liposomal doxorubicin with passive release in IPC has been reported in a number of studies (Sadzuka et al., 1997, 2000; Dadashzadeh et al., 2010; Sugarbaker & Stuart, 2019). Results indicate that the use of liposomes increases tumor concentration of doxorubicin. Moreover, larger-sized liposomes results in slower clearance from the abdominal cavity (Sadzuka et al., 2000). Since liposomes in the abdominal cavity can enter the bloodstream through lymph nodes (Sadzuka et al., 1997), the role of TSLs becomes more important to minimize systemic side effects.

In the literature, there are a number of modeling studies on the use of TSL for drug delivery to a tumor focusing on IV delivery of drugs (El-Kareh & Secomb, 2000; Gasselhuber et al., 2012; Zhan & Xu, 2013). To the best of our knowledge, no mathematical model has yet been reported for IP injection of TSLs, but conventional IPC has been studied in several modeling works (Au et al., 2014; Steuperaert et al., 2017; Shamsi et al., 2018). Au et al. (2014) developed a model for IP delivery of paclitaxel by taking spatially variable parameters into account and considering three different regions for a 2 mm spherical tumor. Steuperaert et al. (2017) introduced a model to study the effects of several different parameters like tumor tissue permeability and tumor size and shape for cisplatin and paclitaxel penetration depths. Shamsi et al. (2018) used magnetic nanoparticles (MNPs) to enhance drug penetration in the tumor tissue in IPC, influenced by a permanent magnet-induced magnetic field. Although their results showed that using drug-coated MNPs can significantly increase drug penetration depth in the tumor, this method can lead to increased side effects by transferring large quantities of the drug to the adjacent normal tissues. Using TSL in IPC can prevent these side effects in addition to improving drug delivery.

In the present work, HIFU-mediated IP delivery of thermosensitive liposomal doxorubicin (TSL-Dox) is evaluated within a mathematical model for the first time. The fluid flow, drug transport, and acoustic and bio heat transfer equations are used in this model. Tumor pathophysiology is reconstructed by considering leaky vasculature, lack of an effective lymph system, and elevated interstitial pressure at the center of tumor. The TSL delivery performance is compared with that of conventional IPC. Further, the effects of parameters including HIFU frequency, TSL size, and vessel wall pore size are investigated. The impact of tumor size on the drug delivery is studied by taking into account three different tumors of 2, 5, and 10 mm in radius. Results of this model are also validated against experimental and numerical studies.

## 2. Materials and methods

**Figure 1.**
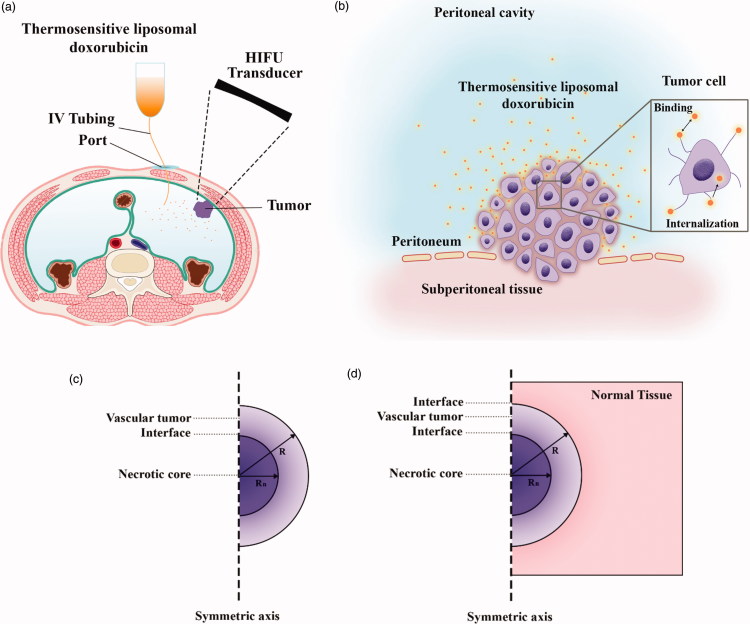
(a) Schematic of high-intensity focused ultrasound-mediated intraperitoneal delivery of thermosensitive liposomal doxorubicin (TSL-Dox delivery); (b) schematic of the drug delivery mechanisms considered in the present study; (c) the geometry corresponding to the fluid flow and mass transport model.

To evaluate the performance of the IP drug delivery system using TSLs, simulations are performed in two parts: first, conventional IP delivery of doxorubicin, and second, HIFU-mediated IP delivery of doxorubicin with TSLs (TSL-Dox delivery). The results of these two simulations are compared to evaluate the performance of a TSL-Dox drug delivery system for IP injection. Figure 1(a) shows a schematic of the IP drug delivery. In conventional IP, the drug is injected into the peritoneal cavity and gradually absorbed into the tumor tissue. In TSL-Dox delivery, on the other hand, TSLs are injected into the peritoneal cavity. A HIFU transducer is used to transfer localized heat and release TSLs inserted into the tumor tissue. The transducer is configured such that its focus area covers the tumor or part thereof. When the drug enters the tumor tissue, it can further penetrate the tumor by means of a convection–diffusion mechanism. The diffusive transfer depends on the diffusion coefficient in the extracellular fluid and the drug concentration. On the other hand, the convective transfer is dependent upon tissue permeability and fluid velocity. Upon tissue entry, the drug can bind to cell surface receptors and then internalize to cancer cells. Figure 1(b) shows the above-mentioned mechanisms for IP drug delivery. The computational domain of the drug transfer equations was considered as a semicircle with the radius *R* (Figure 1(c)). A solid tumor has a spatial heterogeneity. The tumor center may have a necrotic core where there are no blood or lymph vessels, so no fluid exchange occurs with the interstitium. The outer region of the tumor contains rapidly dividing cells and blood vessels. In simulations, therefore, a non-uniform perfusion rate is considered in the tumor center by adding a necrotic core of the radius $R_{n}=$ *R*/2. This model corresponds to experimental observations for non-uniform perfusion (Jain & Ward-Hartley, 1984; Baxter & Jain, 1989, 1990).

**Figure 2.**
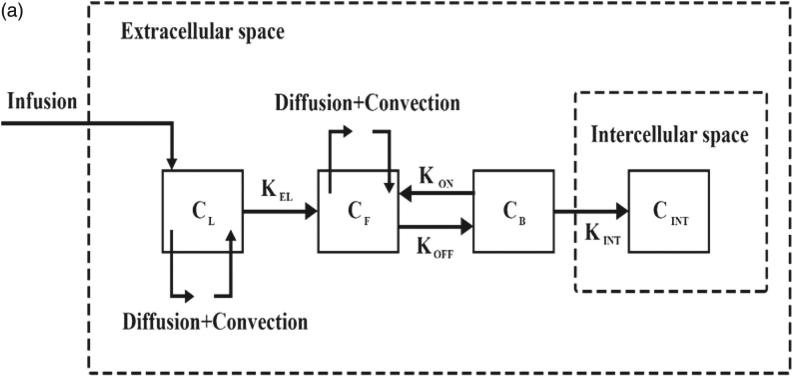
(a) Compartment model of drug transport in intraperitoneal TSL-Dox delivery system.

The mathematical model for conventional IPC includes conservation of mass and Darcy’s equations for the interstitial fluid flow and the convection–diffusion-reaction (CDR) equations for mass transport. Considering the convection and diffusion mechanisms, CDR equations include transport in the interstitium, across the vessels, and such other mechanisms as binding and internalization to cancer cells. Previous studies have detailed the derivation of these equations (Baxter & Jain, 1991; Stylianopoulos & Jain, 2013; Soltani et al., 2014; Sefidgar et al., 2015; Kashkooli et al., 2019). The general mass transfer model is based on compartment models, which are widely used to describe the drug transfer (Soltani et al., 2017). In compartment models, it is assumed that the concentration in each compartment is distributed independently while in the CDR equations, spatial variations of the concentration are also considered by taking convection and diffusion mechanisms into account. In other words, by adding the CDR equations to the compartment model, the concentration distribution in each compartment will be dependent on both space and time. The block-diagram of the model used in the present work is shown in Figure 2(a). The TSL-Dox delivery modeling includes equations that describe the encapsulated doxorubicin transport and release through HIFU heating. These equations include the main equations for fluid flow and CDR equations. Besides, the bio heat transfer equations by considering the HIFU heating are also used to model the TSL drug release. Finally, to quantitatively evaluate the efficiency of both drug delivery systems, a cell survival model is used to calculate the fraction of killed cells (FK).

### 2.1. Conventional IP chemotherapy

Tumor tissue is considered as a porous medium, which is justifiable given that the inter-capillary distance (33–98 μm) is usually 2–3 orders of magnitude smaller than the length scale for drug transfer (Less et al., 1991; Yuan et al., 1995). Therefore, fluid flow in the tumor interstitial space is described using Darcy’s law in a porous medium (Baxter & Jain, 1989):

(1) $$v_{i}=-\kappa \nabla P_{i}$$

where *κ* is the hydraulic conductivity of the interstitium, and *P_i_* and $v_{i}$ are the interstitial fluid pressure (IFP) and velocity, respectively. The steady-state mass conservation equation for an incompressible interstitial fluid is as:

(2) $${\nabla .v}_{i}=\phi_{B}-\phi_{L}$$

where $\phi_{B}$ is the net fluid flow rate per unit volume from blood vessels into the interstitium, and $\phi_{L}$ is the net flow rate per unit volume from interstitium into the lymphatic vessels. $\phi_{B}$ and $\phi_{L}$ are obtained using Starling’s law:

(3) $$\phi_{B}=\;\frac{L_{P}S}{V}\left(P_{B}-P_{i}-\sigma_{s}\left(\pi_{B}-\pi_{i}\right)\right)$$

where *L_P_* is the hydraulic conductivity of the microvascular wall; S/V is the vascular surface area per unit volume; $P_{B}$ and $P_{i},$ respectively, are the intravascular blood pressure and IFP; $\sigma_{s}$ is the average osmotic reflection coefficient for plasma proteins, π*_B_* is the plasma osmotic pressure, and π*_i_* is the interstitial fluid osmotic pressure. Absorption by the lymphatic system, $\phi_{L},$ is related to the pressure difference between the interstitial fluid and lymphatics:

(4) $$\phi_{L}=\;\frac{L_{\mathrm{PL}}S_{L}}{V}\left(P_{i}-P_{L}\right)$$

where *L_PL_* is the hydraulic conductivity of the lymphatic vessel wall, $S_{L}/V$ is the ratio of the surface area of lymphatic vessels to the tumor tissue volume, and $P_{L}$ is the hydrostatic pressure of the lymphatic vessel. Due to the lack of an effective lymphatic system in the tumor tissue, the term $\phi_{L}$ is considered to be zero.

Drug transfer is described by the convection–diffusion equations for free drug in the interstitial fluid. The concentration of free drug in the interstitial fluid ($C_{F}$) is calculated as:

(5) $$\frac{\partial C_{F}}{\partial t}=-v_{i}\nabla C_{F}+D_{F}\nabla^{2}C_{F}-\frac{1}{\varphi }K_{\mathrm{ON}}C_{\mathrm{rec}}C_{F}+K_{\mathrm{OFF}}C_{B}+\Phi$$

where $D_{F}$ is the free drug diffusion coefficient in a porous medium, $C_{\mathrm{rec}}$ is the concentration of cell surface receptors, and *φ* is the tumor volume fraction available to the drug. The $K_{\mathrm{ON}}$ and $K_{\mathrm{OFF}}$ coefficients are the constants of drug binding and unbinding rates, respectively. Φ represents the net total free drug obtained from blood vessels and absorbed by lymphatic vessels calculated as:

(6) $$\Phi =\Phi_{B}-\Phi_{L}$$

where $\Phi_{B}$ is the drug obtained from blood vessels in the tumor and $\Phi_{L}$ is the drug loss through the lymphatic vessels in the tissue unit. Using the pore model (Deen, 1987; Baxter & Jain, 1989, 1990) for trans-capillary exchange, $\Phi_{B}$ and $\Phi_{L}$ are expressed as:

(7) $$\Phi_{B}\;=\left(\phi_{B}\left(1-\sigma_{f}\right)C_{P}+\frac{\mathrm{PS}}{V}\left(C_{P}-C\right)\frac{\mathrm{Pe}}{e^{\mathrm{Pe}}-1}\right)$$

(8) $$\Phi_{L}\;=\phi_{L}C$$

where $C_{P}$ is the drug concentration in the plasma, $\sigma_{f}$ is the filtration reflection coefficient, and *P* is the microvessel permeability coefficient for free drug. As in IP chemotherapy, there is no systematic injection of drug, the term $C_{P}$ can be neglected in the equations. In addition, due to the lack of an effective lymphatic system, the lymph-related term ($\Phi_{L}$) is considered to be zero. Pe is the Peclet number that determines the convection/diffusion ratio through the capillary wall defined as:

(9) $$\mathrm{Pe}=\frac{\phi_{B}(1-\sigma_{f})}{P\frac{S}{V}}$$

The equation related to concentration of cancer cell-bound doxorubicin is as follows:

(10) $$\frac{\partial C_{B}}{\partial t}=\frac{1}{\varphi }K_{\mathrm{ON}}C_{\mathrm{rec}}C_{F}-K_{\mathrm{OFF}}C_{B}-K_{\mathrm{INT}}C_{B}$$

where $K_{\mathrm{INT}}$ is the constant of internalization rate. Finally, the equation for the concentration of internalized doxorubicin will be as:

(11) $$\frac{\partial C_{I}}{\partial t}=K_{\mathrm{INT}}C_{B}$$

### 2.2. HIFU-mediated IP delivery of thermosensitive liposomal doxorubicin delivery

Interstitial fluid flow equations for TSL-Dox delivery include conservation of mass and Darcy’s equations (Equations (1)–(4)). Equations similar to those of 5–8 are also used to describe the encapsulated doxorubicin transport, with an additional equation for the concentration of doxorubicin-containing TSLs. Therefore, the mass transfer equations for the TSL drug delivery system will be as follows:

(12) $$\frac{\partial C_{L}}{\partial t}=-v_{i}\nabla C_{L}+D_{L}\nabla^{2}C_{L}-K_{\mathrm{EL}}C_{L}+\Phi$$

where $C_{L}$ represents TSL-Dox concentration, $K_{\mathrm{EL}}$ is the liposomal drug release constant, and $D_{L}$ is the TSL-Dox diffusion coefficient in the porous medium which is computed by the fiber matrix model described in Fournier (2017) and Shamsi et al. (2018). TSL is designed in such a way to quickly release its contents through heating. The release rates at various temperatures (Table 1) are based on the existing experimental data for a specific liposome formulation (Tagami et al., 2012) and according to the results of fitting on these data. Linear interpolation is used for temperatures between these points. For temperatures above 42 °C, the release rate is considered to be constant.

**Table 1.** Release rates at various temperatures (Tagami et al., 2012).

T(°C)	37	38	39	40	41	42
$k_{\mathrm{EL}}\;(s^{-1})$	0.00417	0.00545	0.01492	0.02815	0.04250	0.05409

The equations for the free, bound, and internalized doxorubicin concentrations are also expressed in Equations (13)–(15), respectively.

(13) $$\frac{\partial C_{F}}{\partial t}=K_{\mathrm{EL}}C_{L}-v_{i}\nabla C_{F}+D_{F}\nabla^{2}C_{F}-\frac{1}{\varphi }K_{\mathrm{ON}}C_{\mathrm{rec}}C_{F}+K_{\mathrm{OFF}}C_{B}$$

(14) $$\frac{\partial C_{B}}{\partial t}=\frac{1}{\varphi }K_{\mathrm{ON}}C_{\mathrm{rec}}C_{F}-K_{\mathrm{OFF}}C_{B}-K_{\mathrm{INT}}C_{B}\;$$

(15) $$\frac{\partial C_{I}}{\partial t}=K_{\mathrm{INT}}C_{B}$$

Tables 2 and 3 represent the values for the parameters used in the model including tissue parameters and solute transport parameters, respectively.

**Table 2.** Parameters for tumor tissue.

Parameter	Definition	Unit	Value	Reference
S/V	Surface area of blood vessels per unit tissue volume	m^–1^	2e4	(Soltani & Chen, 2011)
k	Hydraulic conductivity of the interstitium	m^2^ · Pa^–1^ · s^–1^	3e–14	(Baxter & Jain, 1989)
$L_{P}$	Hydraulic conductivity of the micro-vascular wall	m · Pa^–1^ · s^–1^	2.10e–11	(Sefidgar et al., 2014)
$P_{B}$	Vascular fluid pressure	Pa	2.1e3	(Soltani & Chen, 2011)
$\pi_{B}$	Osmotic pressure of the plasma	Pa	2.7e3	(Baxter & Jain, 1990)
$\pi_{i}$	Osmotic pressure of interstitial fluid	Pa	2e3	(Baxter & Jain, 1990)
$\sigma_{s}$	Average osmotic reflection coefficient for plasma proteins	–	0.9	(Baxter & Jain, 1989)
$a_{f}$	Radius of the tumor matrix fibers	nm	200	(Nacev, 2013)
$r_{p}$	Pore radius of tumor vessels	nm	200	(Stylianopoulos & Jain, 2013)
δ	Vessel wall thickness	μm	5	(Stylianopoulos & Jain, 2013)

**Table 3.** Solute transport parameters used in the simulation.

Parameter	Definition	Unit	Value	Reference
D_eff_	Effective diffusion coefficient	cm^2^ · s^–1^	3.40e–6	(Zhan et al., 2014; Chou et al., 2017)
P	Microvessel permeability coefficient	cm · s^–1^	3.00e–4	(Zhan et al., 2014; Chou et al., 2017)
K_ON_	Constant of binding rate	M^–1^ · s^–1^	1.5e2	(Stylianopoulos & Jain, 2013; Stylianopoulos et al., 2015)
K_OFF_	Constant of unbinding rate	s^–1^	8e–3	(Stylianopoulos & Jain, 2013; Stylianopoulos et al., 2015)
K_INT_	Constant of cell uptake rate	s^–1^	5e–5	(Stylianopoulos & Jain, 2013; Stylianopoulos et al., 2015)
φ	Tumor volume fraction accessible to drugs	–	0.3	(Zhan & Xu, 2013)
C_rec_	Concentration of cell surface receptors	M	1e–5	(Stylianopoulos et al., 2015)
ω	Cancer cell survival constant	m^3^ · mol^–1^	0.6603	(Mpekris et al., 2017)

The nonlinear sound propagation model in a thermo viscose environment is presented as the modified Westervelt equation, which includes the effects of diffraction, absorption, and nonlinearity (Hamilton & Blackstock, 1998):

(16) $$\left(\nabla^{2}-\frac{1}{c^{2}}\frac{\partial^{2}}{{\partial t}^{2}}\right)p+\frac{\delta }{c^{4}}\frac{\partial^{3}p}{{\partial t}^{3}}+\frac{\beta }{\rho c^{4}}\frac{\partial^{2}p^{2}}{{\partial t}^{2}}=0$$

where c is the sound speed, ρ is the density, δ is the acoustic diffusivity, β is the nonlinearity coefficient of the medium, and p is the acoustic pressure. The used acoustic source is a single-element transducer whose parameters are given in Table 4. Since maximum pressure in the focal area is less than 2 MPa in the present study, the error caused by the nonlinear wave effects is less than 5% in the thermal term (Huang et al., 2004), and therefore these effects were neglected.

**Table 4.** HIFU transducer parameters (Huang et al., 2004).

Parameter	Inside diameter	Outside diameter	Focal length	Frequency
Unit	mm	mm	mm	MHz
Value	20.0	70.0	62.64	1.10

In order to couple the pressure field to the temperature field, we need to estimate the thermal energy deposition associated with the absorption of ultrasonic waves. The following equation (Nyborg, 1986) describes the ultrasonic power deposition per unit volume:

(17) $$q=2\alpha_{\mathrm{ABS}}I=\frac{2\alpha_{\mathrm{ABS}}}{\omega^{2}\rho c}〈{\left(\frac{\partial p}{\partial t}\right)}^{2}〉$$

where $\alpha_{\mathrm{ABS}}$ corresponds to the local absorption coefficient, *I* specifies the local acoustic intensity, and the brackets denote time average over one acoustic cycle. In local tumor heating, the tissue temperature can be calculated by solving the energy conservation equation (Pennes, 1948):

(18) $$\rho_{t}c_{t}\frac{{\partial T}_{t}}{\partial t}=K_{t}\nabla^{2}T_{t}-\mathrm{DP}.\rho_{b}c_{b}w_{b}(T_{t}-T_{b})+K_{\mathrm{HIFU}}{.q}_{t}$$

where c is the specific heat, K is the thermal conductivity coefficient, w is the perfusion rate, and $q_{t}$ represents the heat deposition from an external source (HIFU) in the tissue, and *b* and *t* subscripts specify the blood and tissue, respectively. In this equation, DP represents a reduction in the perfusion rate due to heat-induced vessel coagulation, which is assumed to be equal to 1 at normal body temperature and approaches zero by complete vascular shutdown. To model the rate of perfusion reduction due to coagulation, an Arrhenius model is used as follows (Brown et al., 1992; Gasselhuber et al., 2012):

(19) $$\mathrm{DP}=\exp(-\int_{0}^{\tau }A_{f}{e\;}^{\frac{-\Delta E}{\mathrm{RT}\left(\tau \right)\;}}\;d\tau )\;$$

where the parameters $A_{f}$ and ΔE are the frequency factor and the activation energy, respectively, calculated by fitting with the experimental data (Brown et al., 1992).

To reach the ideal temperature range during the simulation, a PI controller is used to adjust the input power based on the temperature set in the temperature region (*T_set_*):

(20) $$K_{\mathrm{HIFU}}=K_{p}\left[T_{\mathrm{set}\;}-T\left(t\right)\right]+K_{i}\int \left[T_{\mathrm{set}\;}-T\left(t\right)\right]$$

where $K_{p}$ and $K_{i}$ are the PI controller parameters. The function of this controller is to prevent tumor site temperature from rising above 43 °C to avoid damage to adjacent normal tissues. Figure 2(b) shows the computational domain of the heat transfer in our model. The values for the acoustic and thermal parameters used in the model are given in Table 5.

**Table 5.** Acoustic and thermal properties.

Parameter	Definition	Unit	Tumor tissue	Normal tissue	Reference
$\nu_{a}$	Ultrasound speed	m · s^–1^	1550	1550	(Sheu et al., 2011)
ρ	Density	kg · m^–3^	1000	1055	(Sheu et al., 2011)
c	Specific heat	J · kg^–1^ K^–1^	3800	3600	(Sheu et al., 2011)
&Kappa;	Thermal conductivity	w · m^–1^ · K^–1^	0.552	0.512	(Sheu et al., 2011)
α_ABS_	Absorption coefficient	Np · m^–1^ · MHz^–1^	8.55	8.55	(Huang et al., 2004)
$\omega_{b0}$	Perfusion rate of blood flow at 37 °C	$s^{-1}$	0.002	0.018	(Vaupel et al., 1989)
R	Universal gas constant	J.mol^–1^ · K^–1^	8.314	8.314	(Gasselhuber et al., 2012)
ΔE	Activation energy for perfusion decrease	J · mol^–1^	6.67e5	6.67e5	(Gasselhuber et al., 2012)
${\mathrm{\&Alpha;}}_{f}$	Frequency factor for perfusion decrease	$s^{-1}$	1.98e106	1.98e106	(Gasselhuber et al., 2012)
$K_{p}$	Controller parameter (proportional term)	–	0.2	0.2	(Gasselhuber et al., 2012)
$K_{i}$	Controller parameter (integral term)	–	0.01	0.01	(Gasselhuber et al., 2012)

### 2.3. Cell survival model

The FK is calculated as $1-s_{F},$ where $s_{F}$ is the fraction of surviving cells. The fraction of surviving cells is calculated using Equation (21) (Mpekris et al., 2017) obtained based on the fitting of an exponential equation on the experimental data for doxorubicin in an *in vitro* study (Kerr et al., 1986).

(21) $$s_{F}=\mathrm{ex}p\left(-\omega \cdot C_{I}\right)$$

where $C_{I}$ is the intracellular concentration of doxorubicin and ω is the fitting parameter as defined in the literature (Kerr et al., 1986).

### 2.4. Initial and boundary conditions

Since the geometry is considered to be symmetrical, half of the computational domain is taken into consideration. Figure 1(c) shows the computational domain for the Darcy and mass transfer equations. The internal boundary condition between the necrotic regions and the tumor tissue is considered as a continuity and is defined for all concentrations and interstitial pressures in Equations (22) and (23):

(22) $$(D_{F}\nabla C+\upsilon_{i}C)\;\&lfloor;\Omega^{-}=(D_{F}\nabla C+\upsilon_{i}C)\;\&lfloor;\Omega^{+}$$

$$C\&lfloor;\Omega^{-}=C\&lfloor;\Omega^{+}$$

(23) $$-k\nabla P_{i}\&lfloor;\Omega^{-}=-k\nabla P_{i}\&lfloor;\Omega^{+}$$

$$P_{i}\&lfloor;\Omega^{-}=P_{i}\&lfloor;\Omega^{+}$$

In IP injection, it is assumed that the drug is present with uniform concentration around the tumor (Steuperaert et al., 2017; Shamsi et al., 2018) so the outer boundary condition for concentration is considered constant and equal to 0.8 mol/m^3^ for both conventional IP chemotherapy and TSL-Dox delivery. The amount of interstitial pressure is also considered constant and equal to zero outside the tumor (Shamsi et al., 2018).

The computational domain for solving the bio heat equation is shown in Figure 2(b). Accordingly, the normal tissue is considered around the tumor and the outer boundary condition is constant thermally and equal to normal body temperature (37 °C). An initial temperature of 37 °C is also considered to solve this equation.

### 2.5. Simulation methods

**Figure 3.**
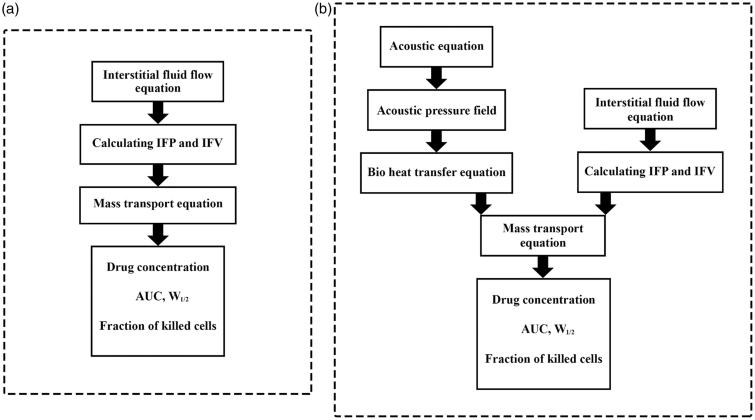
Numerical procedure for (a) conventional IPC modeling and (b) TSL-Dox delivery modeling.

The governing equations, including fluid flow, mass transfer, and bio heat transfer equations, are solved and simulated in COMSOL Multiphysics v5.3a. The duration of IP chemotherapy is considered one hour. Figure 3(a) illustrates a numerical procedure for modeling the conventional IPC. First the interstitial fluid flow equations are solved. The resulting velocity and pressure values are then used to solve the concentration equations.

Figure 3(b) displays the numerical procedure for TSL-Dox delivery equations. By modeling the fluid flow, first the velocity distribution and interstitial pressure are obtained, which are used as input in solving the concentration equations. On the other hand, acoustic pressure is calculated by solving the acoustic equation (Equation (16)), which is used as input in the heat transfer modeling. Since the liposomal release rate in the concentration equations is a function of temperature, the bio heat transfer equation (Equation (18)) couples with the concentration equations (Equations (12)–(15)).

## 3. Results and discussion

The results of TSL-Dox delivery are compared with those of conventional IP chemotherapy. The concentration charts of free drug (*C_F_*), bound drug (*C_B_*), and internalized drug (*C_I_*) are studied and compared in each section. The area under the drug concentration–time curve (AUC) which indicates the amount of extracellular drug available to the tumor is calculated and evaluated for both free drug concentration (*AUC_F_*) and bound drug concentration (*AUC_B_*). Two main criteria are considered for assessing the performance of drug delivery systems:

1. FK values are used as the main parameter for quantitative evaluation of drug delivery efficiency (Equation (21)).
2. The performance of the drug delivery system in enhancing drug penetration depth to the tumor is evaluated by the parameter *W*_1/2_, which is a distance from the tumor outer boundary where the total concentration is equal to 50% of the drug concentration at the tumor border (Au et al., 2014). This parameter is then become dimensionless to compare the drug penetration in tumors with different sizes relative to the tumor radius (*R*), and is investigated as relative half width (*W*_1/2_%).

### 3.1. Conventional IPC

**Figure 4.**
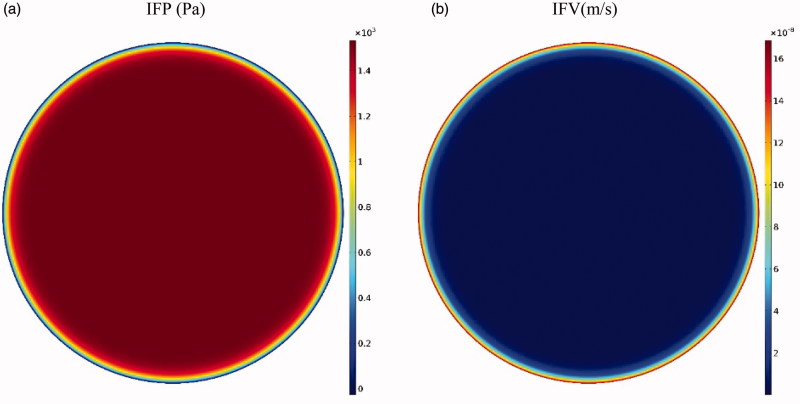
(a) Contours of interstitial fluid pressure and (b) interstitial fluid velocity distribution in the tumor.

The tumor microenvironment has an effective role in the efficiency of drug delivery to the tumor. Higher cell density in tumors leads to a decrease in the tumor tissue permeability compared to normal tissue (Steuperaert et al., 2017). Figure 4(a,b) shows the interstitial pressure and velocity distribution in the tumor. Accordingly, the IFP is uppermost in the tumor center (1533 Pa), except a decrease with steep gradient in a small area near the tumor outer boundary; this high IFP constantly exists in the tumor. According to Darcy’s equation (Equation (1)), as the pressure gradient in a large part of the tumor center is zero, interstitial fluid velocity (IFV) is negligible in this section. As such, due to the high pressure gradient at the tumor exterior, IFV increases with a large gradient and reaches its maximum value (0.17 μm · s^−1^) at the tumor outer boundary (Figure 4(b)). This outward IFV on the tumor outer boundary acts as a barrier for the penetration of antitumor agents during IP chemotherapy.

**Figure 5.**
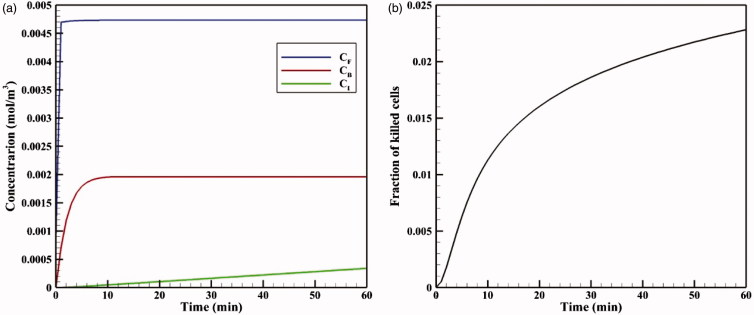
(a) Time profiles of mean free (*C_F_*), bound (*C_B_*), and internalized (*C_I_*) drug concentrations in conventional IPC. (b) Fraction of killed cells (FK) by time in conventional IPC.

Figure 5(a) shows the time profiles for mean concentrations of free, bound, and internalized doxorubicin. As seen, in a very short time after injection in the tumor, *C_F_* reaches its maximum value (0.0046 mol · m^−3^), and then remains constant. The same applies to *C_B_* concentration, with the exception that *C_B_* concentration is maximized with a lower gradient. Contrary to *C_F_* and *C_B_* concentrations, *C_i_* concentration is constantly increasing with a certain gradient. This continuous increase in *C_i_* drug concentration and the non-decreasing trend of *C_F_* and *C_B_* result from the constant drug concentration at the tumor outer boundary during the one-hour injection. Values of *AUC_F_* to *AUC_B_* are 0.292 and 0.113 mol · m^−3^ · s^−1^, respectively, which will be used as reference values for comparing with the results for TSL-Dox delivery. An FK value of 0.022 is observed within 60 minutes after the start of treatment (Figure 5(b)), suggesting a low efficiency of the conventional IPC drug delivery.

**Figure 6.**
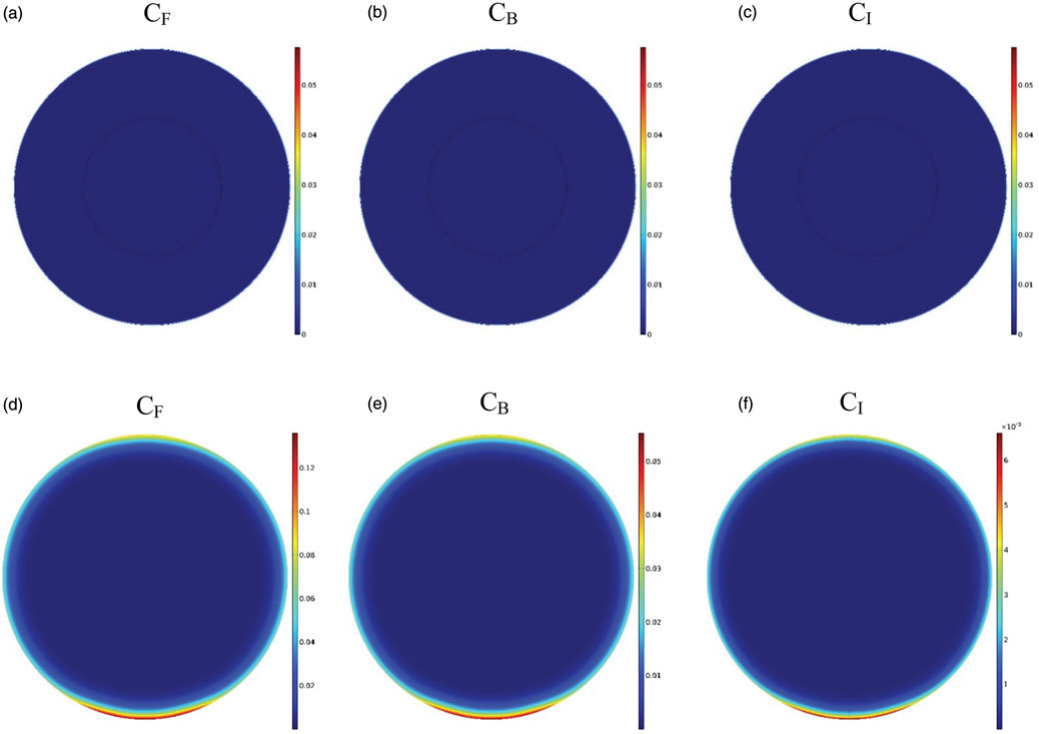
Contours for the concentration distribution of free (*C_F_*), bound (*C_B_*), and internalized (*C_I_*) drug within 60 min for conventional IPC (a–c) and TSL-Dox delivery (d–f).

As depicted in the contours of Figure 6(a–c), the drug penetration in the tumor with IP injection is limited to a very small area of the outer tumor border leaving a large portion of the tumor unavailable to the drug. A value of 30 μm is obtained for *W*_1/2_, which according to the tumor radius of 10 mm, *W*_1/2_% is equal to 0.3%. The results of this section clearly demonstrate one of the major problems with IP injection, that is, a very low drug penetration depth due to the opposing convective flow.

The evaluation of conventional IPC performance indicates a low efficacy of this method according to the low values of *AUC_F_* and *AUC_B_*, FK values, and also the percentage of drug penetration in the tumor (low *W*_1/2_%). In addition to the above, such other challenges as drug side effects and practical obstacles including the rapid drug excretion from the peritoneal cavity predispose the use of this chemotherapy approach to more constraints.

### 3.2. TSL-Dox delivery

The neoplasms resulted in the development of PC varied in size from less than 1 mm to 10 mm. Treatment of larger tumors is more challenging due to the very low drug penetration in the tumor and the risk of disease recurrence (Ansaloni et al., 2015). The results of a large tumor with a radius of 10 mm are discussed in here. Effects of main TSL-Dox delivery system parameters, including the HIFU transducer frequency (*f*) and the TSL size (*a*), were studied by changing these parameters in the clinically reasonable ranges. The effect of tumor size in the drug delivery is examined by analyzing the results of two small and medium tumors with 5 mm and 2 mm in radius, respectively.

**Figure 7.**
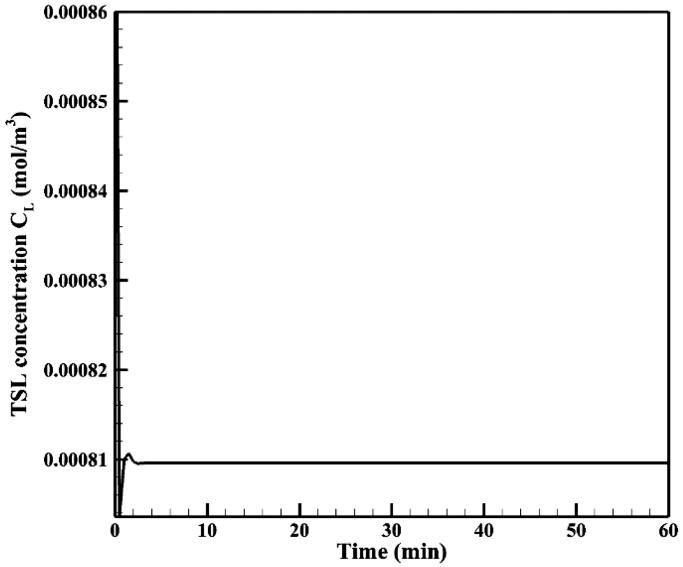
Mean TSL concentration (*C_L_*) in the tumor.

Figure 7 shows the time profile of mean TSL-Dox concentration (*C_L_*) in the tumor. Mean TSL-Dox concentration in the tumor increases after injection of TSL-Dox into the peritoneal cavity within a very short time lapse. By applying heat through the HIFU transducer and with the rising temperature, doxorubicin is released from TSLs at a high rate and *C_L_* drops with a sharp gradient. After this stage and with continuous heat transfer, an equilibrium is established between the entry of TSLs in the tumor and their heat-induced release, thereby, *C_L_* reaches a constant equilibrium within 60 minutes (Figure 7).

**Figure 8.**
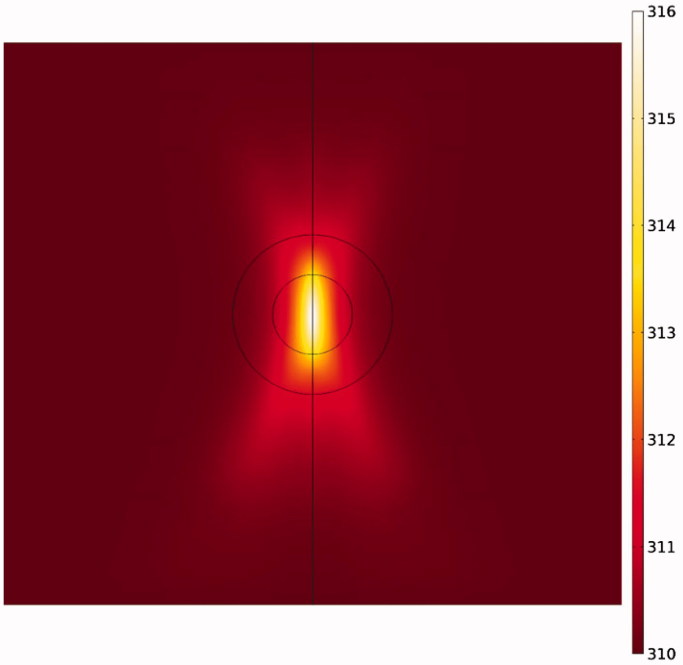
Contour of temperature distribution after HIFU heating with an operating frequency of 1 MHz.

Due to the elliptic feature of the heated focal region (Figure 8), the concentration distribution in the tumor is also asymmetric (Figure 6(d–e)), with the upper and lower tumor areas containing the highest drug concentrations. In other words, because TSLs enter the tumor from the outer tumor boundary, and due to the presence of an outward convection flow, this penetration is limited to areas close to the tumor border. Rising temperatures in the central regions of the tumor, where liposome concentration is zero or close to zero, have no effect on the drug delivery process. In contrast, it is important to increase the temperature in the tumor border or near the border due to the accumulation of TSLs in these areas leading to TSL drug release. Hence what determines the concentration distribution in the tumor is the distribution of temperature near the tumor borders. Since the focal region is elliptic in HIFU-mediated heating, the tumor borders lying at the elongated side of the ellipse, as the upper and lower tumor areas, experience liposomal release at high rates. The rest of tumor boundaries, however, will have low drug release as not being adequately heated.

**Figure 9.**
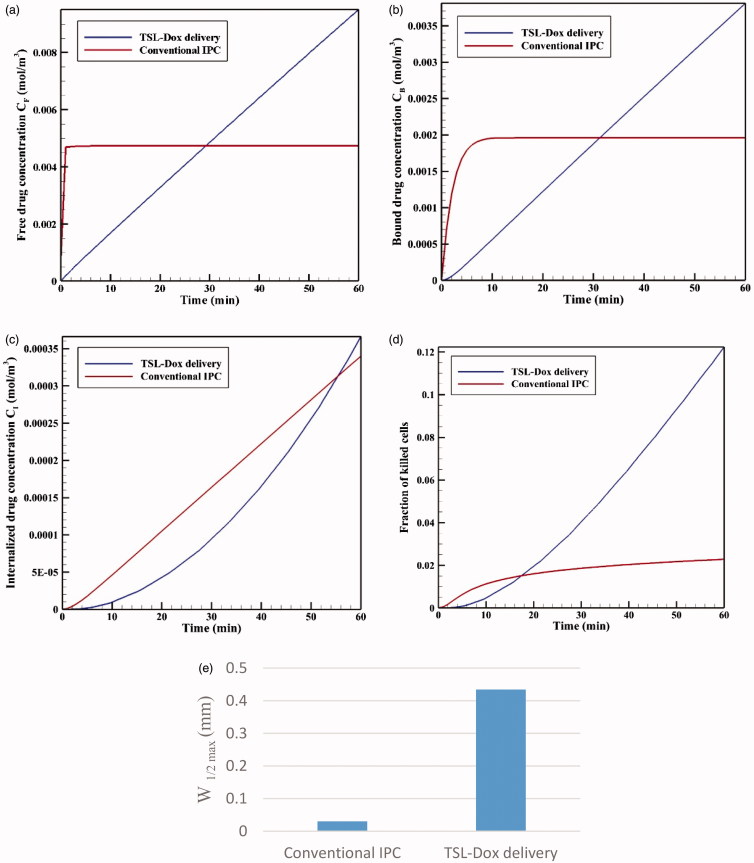
Time profiles for mean concentrations of (a) *C_F_*, (b) *C_B_*, (c) *C_I_*, (d) fraction of killed cells (FK) as a function of time for both conventional IPC and TSL-Dox delivery. (e) Maximum drug penetration depth into the tumor (*W*_1/2max_) in two methods of TSL-Dox delivery and conventional IPC.

The time profiles of mean *C_F_*, *C_B_*, and *C_I_* values are shown in Figure 9(a–c), respectively. The analysis of these graphs shows that at initial minutes of the injection, the values of all three concentrations are lower in TSL-Dox delivery than the corresponding values for conventional IPC, but it surpasses over time. In fact, because particles of free drug are injected directly at the beginning of conventional IPC, these particles have a greater accumulation in the tumor due to their smaller size. Ultimately, *C_B_* and *C_I_* values will also be greater than those of TSL-Dox delivery. However, since free doxorubicin supply is related to release from TSLs, and because there is always a constant mean concentrations of these carriers in the tumor during injection (Figure 7), the continuous release of free drug raises the concentration of free doxorubicin continuously. Over time, therefore, the concentration values in TSL-Dox delivery system approach the corresponding values for conventional IPC and ultimately exceed these levels.

Figure 9(d) illustrates the time profiles for FK values in conventional IPC and TSL-Dox delivery. Although FK is higher for conventional IPC at early stages, FK values in TSL-Dox delivery gradually exceed that of conventional approach at times over 17 minutes. According to this chart and concentration graphs (Figure 6(a–c)), TSL-Dox delivery method has a much higher efficiency than conventional IPC.

Drug penetration depths in the two drug delivery methods (Figure 9(e)) indicate a significant penetration depth increase in TSL-Dox drug delivery. A comparison of *W*_1/2max_ values shows that drug penetration depth in TSL-Dox delivery was 14.5 times higher than that of the conventional method. In conventional IPC, chemotherapy drugs after entering the tumor immediately bind to cancer cells within the tumor boundaries and, therefore, cannot further penetrate the tumor. In TSL-Dox delivery system, on the other hand, as the drug is transferred by carriers, the particles have the opportunity to further penetrate the tumor. In fact, there is a competition for a free drug between rapid diffusion and drug binding to nearby cancerous cells (Mok et al., 2009; Schmidt & Wittrup, 2009). As a result, free drug particles in conventional IPC bind to cancer cells more effectively, so that there is lesser penetration in this method than the use of TSL.

#### 3.2.1. Effect of frequency

**Figure 10.**
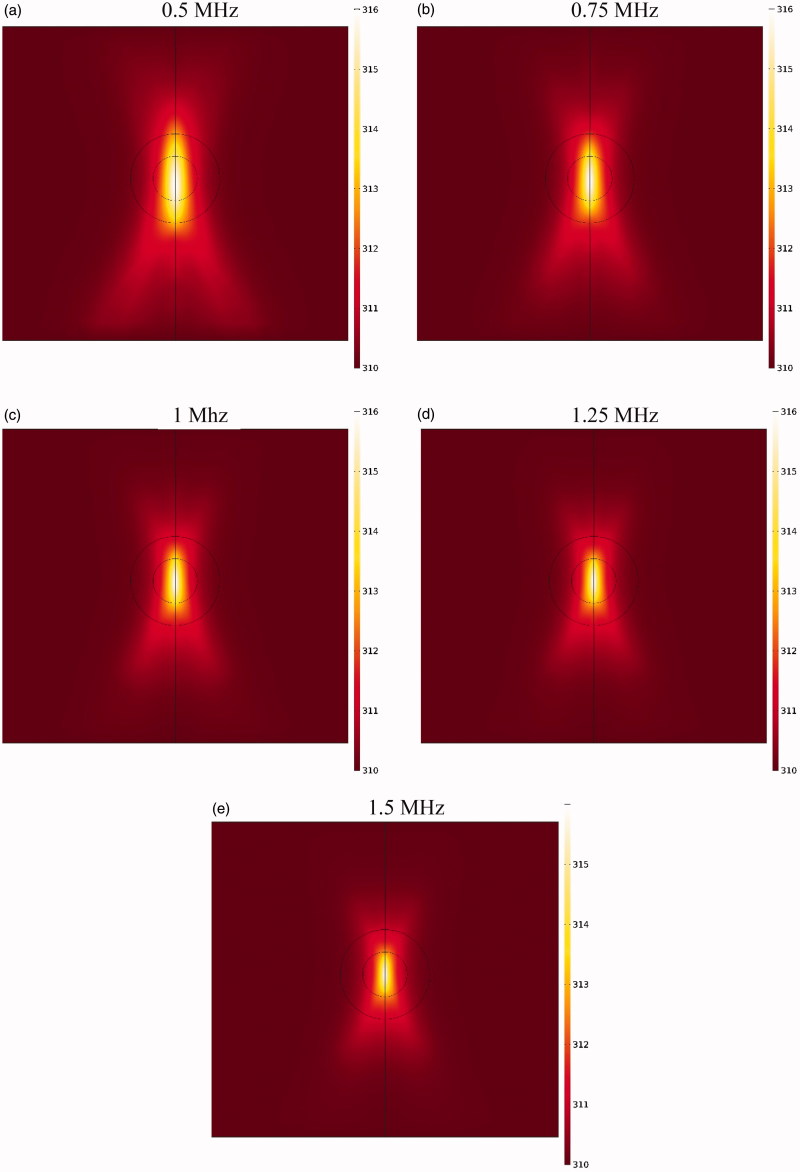
Temperature distribution contours at different operating frequencies of HIFU heating.

If the HIFU focal area is such that it affects an area outside the tumor, it will release the drug into the peritoneal fluid raising the risk of side effects. To achieve the lowest risk of side effects, therefore, the focal area should be adjusted so that the temperature at the tumor boundaries and its exterior does not reach the temperature range (nearly 42 °C) of a high liposomal release rate. Figure 10(a–e) shows the contours of temperature distribution in the tumor for various operating frequencies of 0.5, 0.75, 1, 1.25, and 1.5 MHz. HIFU focus was set on the tumor center. The figure clearly shows that changing the HIFU transducer frequency results in a change in the focal area size, and that the higher the frequency, the smaller the focal area. Therefore, changes in this frequency can potentially affect drug delivery to the tumor. For the lowest frequency (0.5 MHz, Figure 10(a)), this area also encompasses a part of the tumor border, while the focal area is drawn into the tumor boundaries at 1 MHz (Figure 10(c)) and ultimately lies close to the necrotic core at 1.5 MHz (Figure 10(e)).

**Figure 11.**
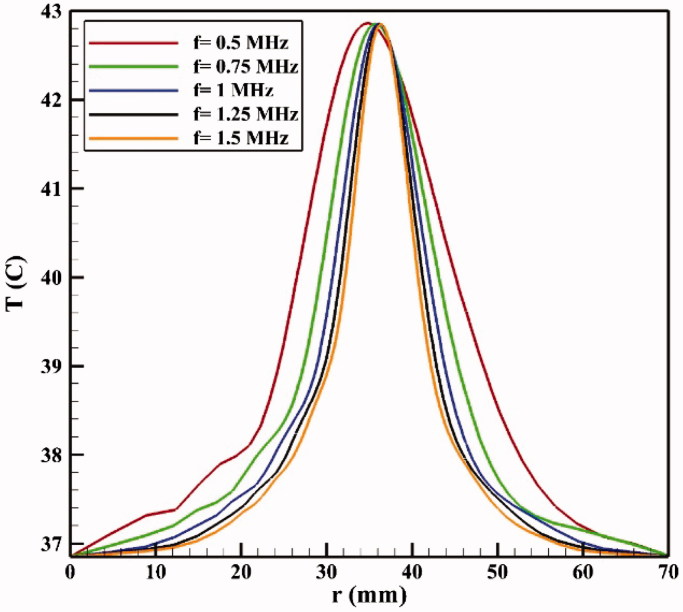
Temperature distribution profiles along the vertical line passing through the tumor center (symmetric axis in Figure 1(d)) for different frequencies.

Figure 11 shows the temperature distribution profiles along the vertical line passing through the tumor center at various frequencies. The analysis of these profiles reveals that the tumor border temperature is 41.82 °C at a frequency of 0.5 MHz, with a high TSL release rate at this temperature (Table 1). In addition, outside the tumor area, the temperature is also very close to maximum temperature of TSL release (42 °C) in areas close to the upper and lower tumor boundaries. This means high release rates of TSLs within the peritoneal cavity possibly leading to side effects. With narrowing of the focal area at a frequency of 0.75 MHz, the temperature reached 40.51 °C at the tumor border, and the release rate decreased at the tumor border compared to that at a frequency of 0.5 MHz. In such a condition, the temperature is still close to that of high TSL drug release in parts of the tumor exterior, and the use of this frequency will still result in the risk of side effects. At a frequency of 1 MHz, the focal area is completely drawn into the tumor borders. In this state, the temperature is 39.61 °C on the tumor boundary; hence, there will be a relatively low liposomal release rate at the tumor border and its exterior area compared to the two previous frequencies. Finally, for two frequencies of 1.25 and 1.5 MHz, the temperature is 39.08 and 38.92 °C, respectively, on the tumor boundary, indicating a low release rate on the tumor border and its exterior area.

**Figure 12.**
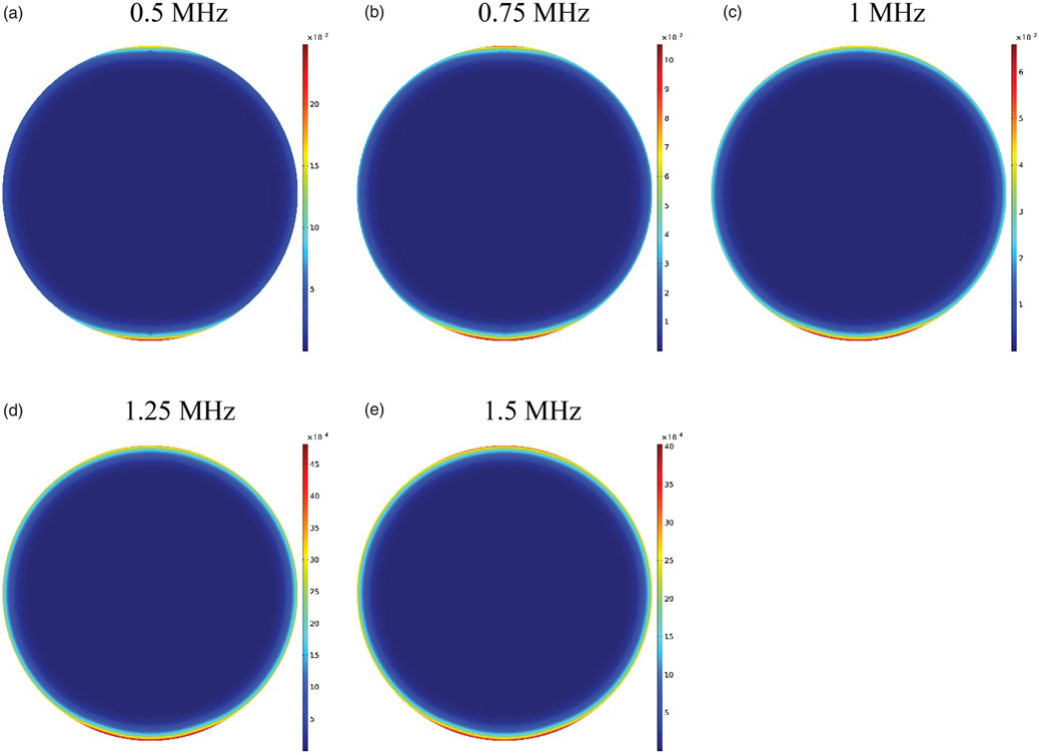
Contours of the concentration distribution of *C_I_* drug at five examined frequencies within 60 minutes after drug delivery.

**Figure 13.**
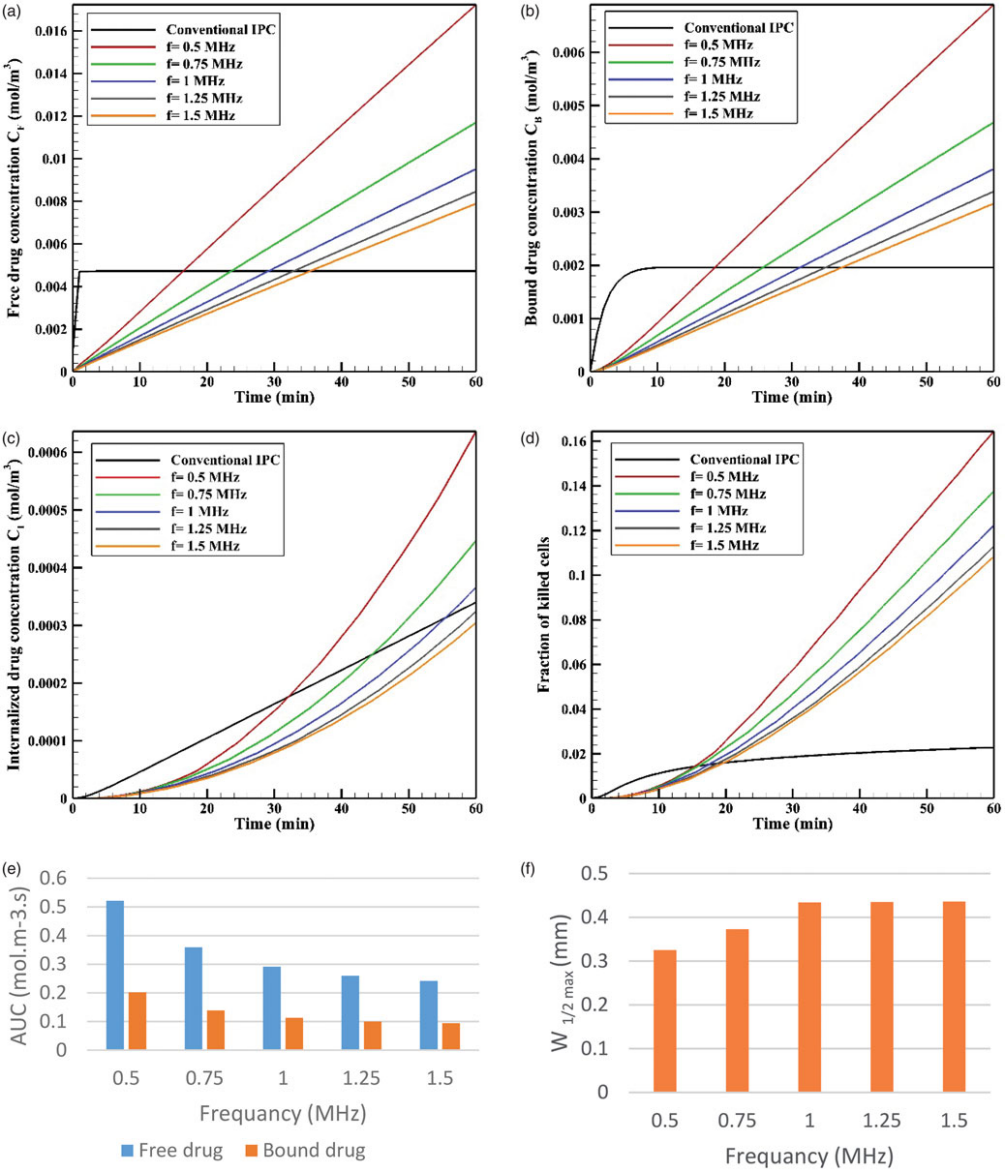
Concentrations of (a) *C_F_*, (b) *C_B_*, and (c) *C_I_*. (d) The fraction of killed cells at different tested frequencies. (e) Comparison of *AUC_F_* and *AUC_B_* at different tested frequencies. (f) Maximum drug penetration depth (*W*_1/2max_) into the tumor in TSL drug delivery at the tested frequencies (*t* = 60 min).

Figure 12(a–e) shows *C_I_* drug distribution at five different frequencies after an hour of drug delivery. A careful examination of these contours suggests that at lower frequencies where the focal area has a wider range, drug release occurs in most of the tumor and is not limited to the tumor upper and lower areas. In addition, a comparison of the profiles in Figure 11 reveals that the lower the frequency, the larger the tumor region undergoing high temperatures, leading to increased rate of liposomal drug release. Overall, it can be expected that more drug is released into the tumor at lower frequencies. This is also confirmed by the examination of average concentrations (Figure 13(a–d)). At 0.5 MHz, the highest mean concentrations of free, bound, and internalized drug occur at all the times (Figure 13). For better analysis of this phenomenon, the AUCs were calculated for extracellular concentrations (Figure 13(e)). The results showed that by increasing the frequency (0.5–1.5 MHz), the values of *AUC_F_* and *AUC_B_* dropped from 0.522 to 0.242 mol · m^−3^ · s^−1^, and from 0.202 to 0.092 mol · m^−3^ · s, respectively.

The time profile of FK values at five examined frequencies is shown in Figure 13(d). As expected, FK values are higher at lower frequencies at all times; in fact, higher drug concentrations available to the tumor, followed by an increase in *C_I_* at lower frequencies, make chemotherapy more effective at these frequencies. According to Figure 13(d), the FK values surpass those of conventional IP chemotherapy at all five examined frequencies after 20 minutes of the treatment onset, with increasing levels over time. The FK value is 0.17 at a frequency of 0.5 MHz within 60 minutes after injection, which is approximately 8.5 times that of the conventional injection at the same time. At the largest tested frequency (1.5 MHz), the FK value was equal to 0.1 at 60 minutes, showing a fivefold increase compared to conventional IP chemotherapy.

Although the rising mean concentrations of AUC and FK at lower frequencies has a positive indication of an effective drug delivery to the tumor, it should be noted that the decreasing frequency and enlargement of the focal region amplify the probability of high liposomal release rates in the peritoneum cavity. To achieve the lowest risk of side effects, therefore, this increase in frequency should be commensurate to the tumor geometry and dimensions. Based on the results obtained in the previous section for presently examined tumor, frequencies of 0.5 MHz and 0.75 MHz have a higher risk of unwanted high-dose liposomal release in the peritoneum among the tested frequencies.

To compare the effects of different frequencies on the drug penetration depth, values for the five examined frequencies are shown in Figure 13(f). It suggests that increasing the frequency from 0.5 MHz to 1 MHz raises the penetration depth, which is attributable to the liposome release near the tumor border occurring at lower frequencies. In other words, because the temperature is close to 42 °C in the tumor border at a frequency of 0.5 MHz, the drug is released from the liposome at high rates and, at the same time, the outer tumor boundary binds to the cancerous cell. On the other hand, as the temperature is lower near the tumor border at 1 MHz, the drug is released at lower rates near the border, and more TSLs remain available for penetration into the tumor interior. Another point in Figure 13(f) is that increasing the two frequencies of 1.25 and 1.5 MHz has not led to further increase in the penetration depth compared to 1 MHz frequency, which might have resulted from the focus of the heated area on the necrotic region. In fact, since liposomal penetration is absent in the necrotic region, the heating focus on this tumor area does not play a role in improving drug delivery. Overall, frequencies close to 1 MHz can be considered more appropriate for the studied tumor as the highest penetration depth and FKs occur at this frequency, with a low risk of drug release in the peritoneal cavity.

#### 3.2.2. Effect of liposome size

**Figure 14.**
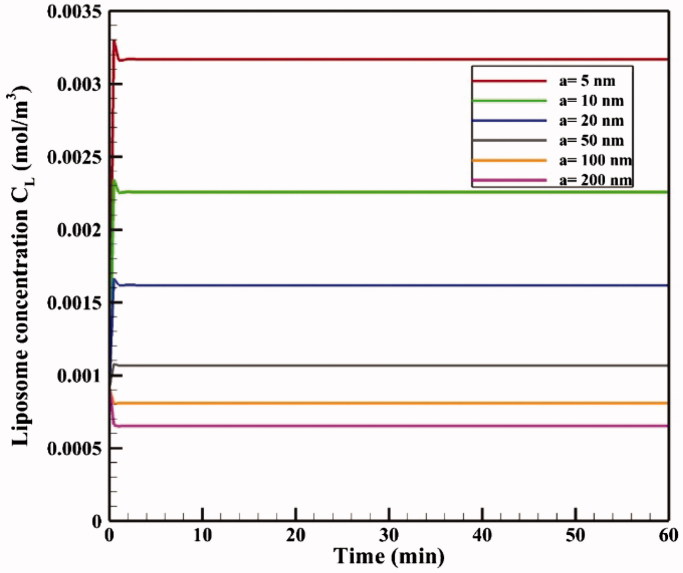
Mean TSL concentrations in the tumor for different sizes of TSL.

The effect of TSL size on drug delivery efficiency is investigated in liposomes with dimensions of 5–200 nm in diameter. The size of TSLs determines their diffusion coefficients in the interstitial space and, thus, may potentially affect drug delivery. Figure 14 shows mean tumor TSL concentrations for different sizes of liposomes as a function of time. Accordingly, a decrease in liposome size increases average tumor concentrations of liposome because smaller liposomes can pass through the tumor. This increase in concentration leads to elevated levels of *C_F_*, *C_B_*, and *C_I_* drug followed by boosted chemotherapy efficacy, so that changing liposomal size has a terrific effect on FK levels. The FK value is approximately 0.11 for a 200 nm liposome at *t* = 60 min, but it is doubled to 0.24 by using a 5 nm liposome. The *AUC_F_* and *AUC_B_* values also increased considerably with a decrease in TSL size. For example, the *AUC_F_* value is 0.236 mol · m^−3^ · s^−1^ for a 200 nm TSL, but this amount rises to almost five times (1.142 mol · m^−3^ · s^−1^) with a TSL size of 5 nm. In addition, TSL size simulation results showed no significant impact of TSL size on drug penetration depth, with a penetration value of 0.43 for all the liposome sizes under the same thermal conditions. Considering that the depth of drug penetration into the tumor is generally very limited in IP injection, even for the direct injection of doxorubicin in conventional IPC, the use of TSLs with much larger sizes than free doxorubicin cannot significantly reduce the penetration depth.

#### 3.2.3. Effect of vessel wall permeability

**Figure 15.**
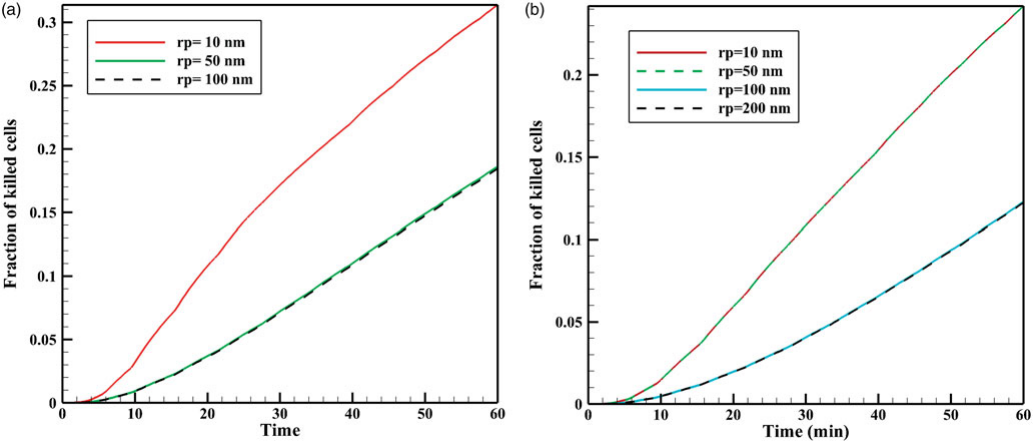
FK as a function of time for different vessel wall pore sizes: (a) 20 nm TSL and (b) 100 nm TSL.

During IP injection, low molecular weight drugs can rapidly absorbed by capillaries and enter the circulatory system. This, in addition to the loss of the drug available to the tumor, can add to the side effects of chemotherapy. Therefore, in addition to the effect of TSL size on the drug transfer in the interstitial space, drug transfer through the vessel wall is also affected by the ratio of TSL size to vessel wall pore size ($r_{p}$) as the parameter representing vessel permeability (Stylianopoulos et al., 2015). In Figure 15(a,b), FKs as a function of time are plotted for various sizes of vessel pores with TSLs of 20 nm and 100 nm. As the vessel wall transfer depends on the TSL size and the vessel wall pore size, both the pore and the liposome dimensions affect the chemotherapy efficiency. According to the results (Figure 15(a,b)), the amount of FK decreases significantly when the vessel pore size is larger than the TSL size. As shown in Figure 15(b), for a 100 nm liposomal size at 60 minutes, the FK value is 0.12 when the vessel pore size is 200 or 100 nm, but it is 0.24 for a vessel size smaller than 100 nm. Thus, increasing vessel pore diameter leads to elevated loss of TSL through the vessel, thereby, reducing the treatment efficiency. Although based on results of pervious section, a decrease in TSL size can improve the treatment efficacy in this method, so the TSL dimensions should be reduced considering the vessel wall pore size. Additionally, it should be noted that experimental results (Sadzuka et al., 2000) demonstrate that TSL with a larger size has a longer residence in peritoneum cavity and will be available to the tumor for a longer time period.

#### 3.2.4. The effect of tumor size

**Figure 16.**
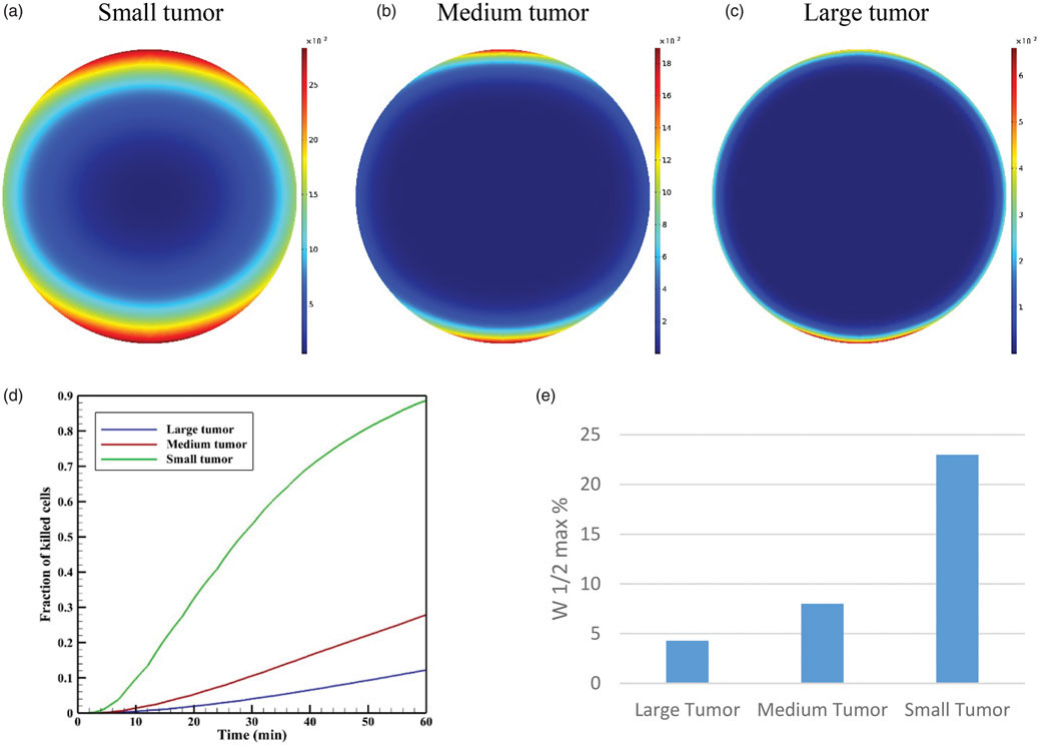
Concentration of *C_I_* in three tumors: (a) small, (b) medium, and (c) large at *t* = 60 minute. The TSL size is 100 nm. (d) FK values as a function of time in TSL-Dox delivery with different tumor sizes. (e) The effect of tumor size on the drug penetration into the tumor.

The results presented in previous sections were for a large tumor with a radius of 10 mm. In this section, the results for two medium and small tumors (5 and 2 mm in radius) are compared with that of the large tumor. Figure 16(a–c) shows the contours of *C_I_* drug for three small, medium, and large tumors for a TSL size of 100 nm at *t* = 60 min. The figure displays that TSL-Dox delivery for a small tumor has a better drug distribution and penetration than the medium and large tumors.

The values of *AUC_F_* for large, medium, and small tumors were calculated as 0.292, 0.855, and 6.967 mol · m^−3^ · s^−1^, indicating a significant increase with reductions in the tumor size. The values of *AUC_B_* also indicate a significant increase in this parameter by reducing the tumor size from 0.113 mol · m^−3^ · s^−1^ for a large tumor to 2.695 mol · m^−3^ · s^1^ for a small tumor. It can, therefore, be concluded that the extracellular drug concentration available to the tumor is greater in smaller tumors. Figure 16(d) shows FK values as a function of time for three different tumor sizes. It is seen that the treatment efficiencies have significantly increased with decreasing tumor size. FK values for medium and small tumors are 0.28 and 0.88, respectively, at *t* = 60 min showing a considerable rise compared that of 0.12 obtained for the large tumor.

In addition, Figure 16(e) compares the relative drug penetration depth for different tumor sizes with a TSL of 100 nm. The relative penetration is 23% in the small tumor, which is approximately four times that of medium tumors and nearly six times that of large tumors. Therefore, smaller tumors have generally a better status than the larger tumors in terms of drug availability to the tumor and penetration depth.

### 3.3. Validation

This section deals with the validation of the numerical simulations. Since the simulation of this problem is obtained from solving different physics and various equations, including the Darcy equation to find the pressure and velocity distributions, mass transfer equations to find the distributions of free, bound, and internalized drug, the Westervelt equation to find the acoustic pressure, and bio heat equation to calculate the temperature. Therefore, it is necessary to verify each physics and to compare with results of various references.

#### 3.3.1. Interstitial fluid pressure and interstitial fluid velocity validation

**Figure 17.**
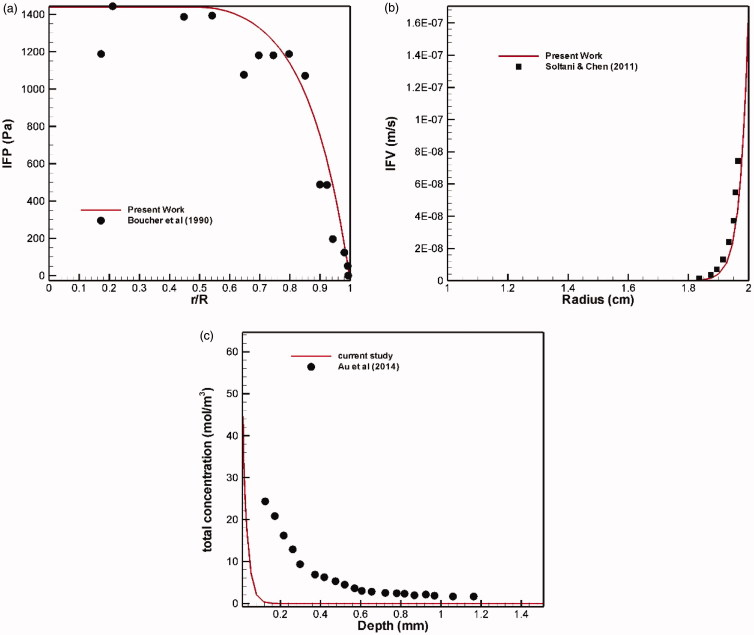
Validation of (a) interstitial fluid pressure (IFP) and (b) interstitial fluid velocity (IFV). (c) Concentration profile in terms of penetration depth after six hours of injection.

One of the most important parts of this modeling is to find the distribution of interstitial pressure and velocity, which is obtained by solving the Darcy equation according to Equation (1). To this end, a comparison is made between the radial distributions of interstitial tissue pressure with experimental works (Boucher et al., 1990) in the same conditions (Figure 17(a)) showing a good agreement between experimental and theoretical results. Also, mean interstitial velocity is compared with the theoretical values obtained by Soltani & Chen (2011), which shows a remarkable agreement (Figure 17(b)).

#### 3.3.2. Verification of the interstitial concentration distribution

Au et al. (2014) examined chemotherapy on a murine tumor using paclitaxel by the conventional IP method. Total concentration profile in terms of penetration depth after a six-hour period is shown in Figure 17(c). The tumor radius was *R* = 2 mm and the drug concentration was *C_tot_* = 45 mM at the tumor border. There is a significant difference between the two charts, but there is a similar behavior qualitatively. This difference may be due to different tissue and drug properties.

#### 3.3.3. Verification of acoustic pressure distribution

As mentioned above, an ultrasonic device is used to heat the tumor tissue locally to increase the liposomal release rate with the transducer used in the work of Huang et al. (2004). They used a single-element, piezoceramic spherical transducer (Models H-102 and H-101, Sonic Concepts, Woodinville, WA) with a central hole of 20 mm in diameter. The transducer has a focal length of 62.64 mm, an aperture of 70 mm, and an operating frequency of 1 MHz. Supplementary Figure S1(a,b) shows the dimensionless acoustic pressure distribution in two axial and radial directions inside the tumor, which is in well agreement to the experimental results of Huang et al. (2004). In these diagrams, the absolute acoustic pressure becomes dimensionless with maximum pressure at the focal point.

Supplementary Figure S2(a) shows the acoustic pressure distribution obtained from solving the Westervelt equation, as well as the distribution of acoustic intensity (Supplementary Figure S2b). The acoustic pressure value is equal to 0.9 MPa in the focal area. As the distance from the focal area increases, the amounts of acoustic pressure and intensity decrease as well.

#### 3.3.4. Thermal verification

By increasing both the local acoustic pressure and acoustic intensity in the focal area, the amount of heat generated in this area behaves according to Equation (17). Therefore, it is expected that the temperature in this area is maximal. Huang et al. (2004) also plotted the temperature at the focal point in terms of elapsed time. They turned off the ultrasound device after a second. The ascending temperature rise stops in the first second, after which the temperature falls. As shown in Supplementary Figure S3, the results of our simulation for temperature at the focal point has a good agreement with presented values of Huang et al. (2004), providing the performance of heat transfer solver in the presence of HIFU heat source.

## 4. Conclusions

IP chemotherapy is commonly used as a locoregional treatment for patients with PC, often originated from ovarian or colorectal carcinoma. Although IP chemotherapy has been promising for certain cases of patients with colorectal cancer, the inadequate drug delivery to the tumor and its side effects have strongly restricted the clinical use of this drug delivery method. In this study, a new drug delivery strategy is proposed based on intraperitoneal (IP) injection of TSL-Dox in combination with mild HT induced by high intensity focused ultrasound (HIFU). A mathematical model is developed to assess the feasibility of the proposed drug delivery system. FK values and the drug penetration depth into the tumor are considered as the two main criteria for evaluating the efficacy of the proposed drug delivery system. Various factors, including the effect of HIFU frequency, TSL size, vessel wall pore size, and the tumor size can influence the FK value and drug penetration depth. A set of parametric studies is conducted to examine the impacts of the above-mentioned parameters. The following conclusions are drawn based on our results:

1. The use of TSL-Dox delivery in IP injection is much more effective than conventional IP chemotherapy, so that using 100 nm TSLs and thermal condition created with 1 MHz HIFU frequency in large tumors lead to 14.5 times increase in the drug penetration depth and more than six times elevation in FK values within one hour after the injection.
2. The effect of tumor tissue temperature on the performance of the proposed drug delivery system is evaluated by changing the HIFU frequency in the range of 0.5–1.5 MHz. The results show that concentration distribution in the tumor is determined by temperature distribution near the tumor boundaries. As the frequency decreases, the amounts of FK increases, however, decreased frequency led to the enlargement of the heated focal point. Therefore, it should be noted that with excessive frequency reduction, the heated region may encompass the tumor boundaries and cause the release of TSLs in the peritoneal fluid. In addition, the results show an improvement in drug penetration by increasing the HIFU frequency up to 1 MHz. With further increase in frequency the focal area will be limited to the necrotic core of the tumor and the depth of penetration remains unaffected. According to the results of the frequencies studied, frequencies close to 1 MHz have the highest treatment efficiency and, at the same time, the lowest risk of unintentional dug release in the peritoneal cavity.
3. The effect of TSL size (5–200 nm) is investigated on the performance of the proposed drug delivery system. The results show that decreased TSL sizes could enhance the FK values and the treatment efficiency. FK values are 11% and 24% when using 200 nm and 5 nm TSLs within an hour after treatment while the corresponding values for conventional IPC is about 2%. Also, the size of TSLs had no significant effect on the drug penetration depth.
4. The results show that the vessel wall pore size in the tumor could have a high impact on the efficiency of this method. If the size of TSLs is smaller than that of vessel wall pore size, a marked amount is lost through these vessels, thereby, decreasing the treatment efficiency. These drugs increase the risk of chemotherapy systematic side effects by entering the bloodstream.
5. The use of TSL-Dox delivery method has a far greater effect on smaller tumors. In a small tumor with a radius of 2 mm, the FK value reaches 0.88 at one hour after the injection, indicating a high efficiency of the drug transfer system. In addition, the drug penetration depth into the tumor is significantly higher for smaller tumors.

The results are of high accuracy and reliability as verified by various numerical and experimental tests.

## Supplementary Material

Supplemental Material
