# Supplementary material for: Numerical modeling of high-intensity focused ultrasound-mediated intraperitoneal delivery of thermosensitive liposomal doxorubicin for cancer chemotherapy
Source: Drug Deliv. 2019 Sep 16;26(1):898–917. doi: 10.1080/10717544.2019.1660435 (PMC6758722; doi:10.1080/10717544.2019.1660435)
Supplement: Supplemental Material [file IDRD_A_1660435_SM7610.docx]

**Numerical modeling of** **high-intensity focused ultrasound-mediated intraperitoneal delivery of thermosensitive liposomal doxorubicin for cancer chemotherapy**

Mohsen Rezaiean^1^, Amir Sedaghatkish^2^, M. Soltani^1,3,4,5,6, *^

^1^ Department of Mechanical Engineering, K. N. Toosi University of Technology, Tehran, Iran

^2^ Department of Mechanical Engineering, Isfahan University of Technology, Isfahan, Iran

^3^ Advanced Bioengineering Initiative Center, Computational Medicine Center, K. N. Toosi University of Technology, Tehran, Iran

^4^ Department of Electrical and Computer Engineering, University of Waterloo, ON, Canada

^5^ Centre for Biotechnology and Bioengineering (CBB), University of Waterloo, Waterloo, Ontario, Canada

^6^ Cancer Biology Research Center, Cancer Institute of Iran, Tehran University of Medical Sciences, Tehran, Iran

* Corresponding author:

Email address: [msoltani@uwaterloo.ca](mailto:msoltani@uwaterloo.ca)

First author email address:

[mohsenrezaeian@email.kntu.ac.ir](mailto:mohsenrezaeian@email.kntu.ac.ir)

Second author email address:

[a.sedaghat@me.iut.ac.ir](mailto:a.sedaghat@me.iut.ac.ir)

**Supplemental Information**

Figures related to the acoustic pressure distribution and thermal verification is presented. The reader is referred to **Section 3.3.3** for acoustic verfication **and Section 3.3.4** for thermal verification in the main text for the discussion on the figures.

| 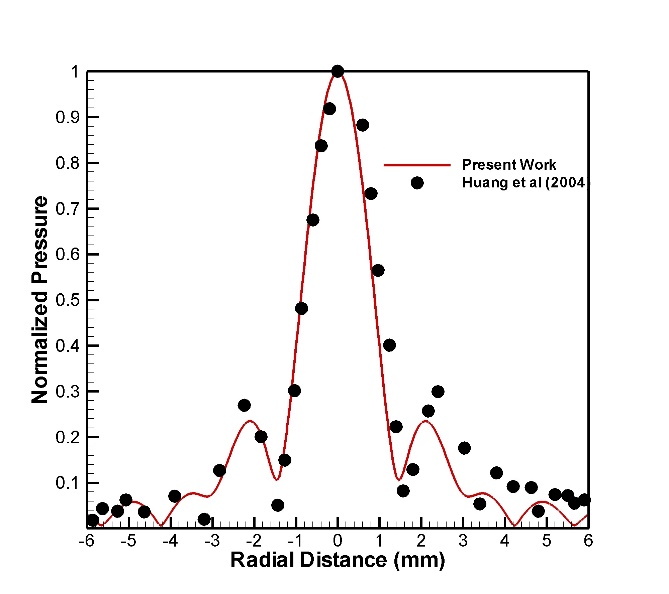 | 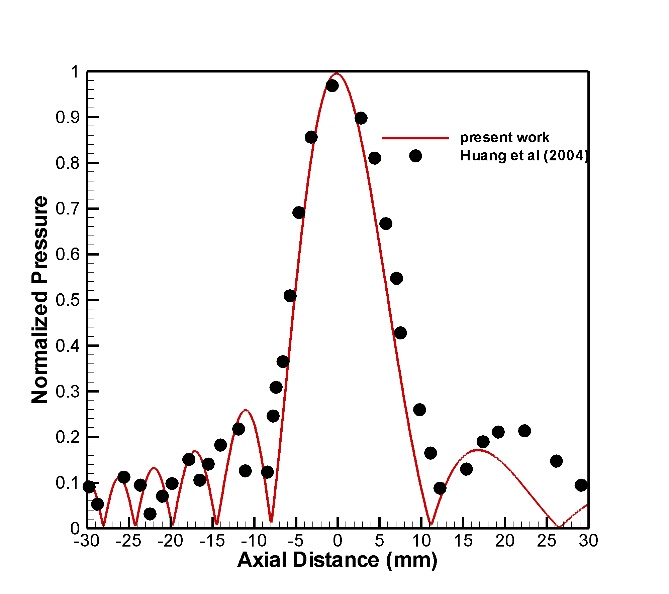 |
| --- | --- |
| **Radial direction(b)** | **(a) Axial direction** |
| **Fig S1. Acoustic pressure in two direction (a) axial (b) radial** | |

| 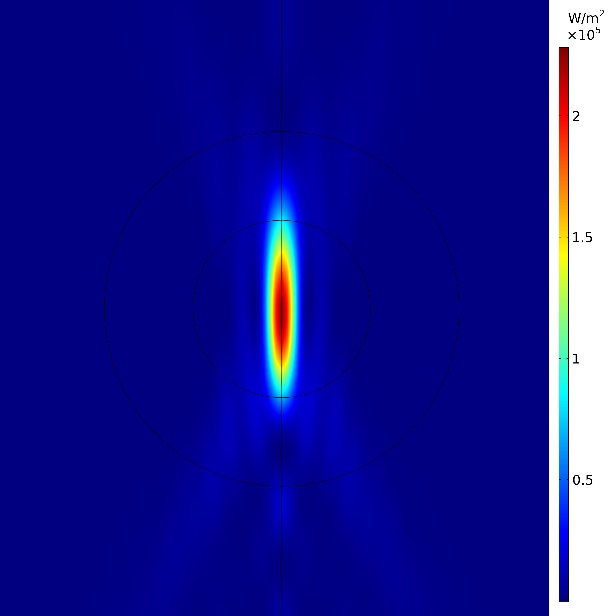 | 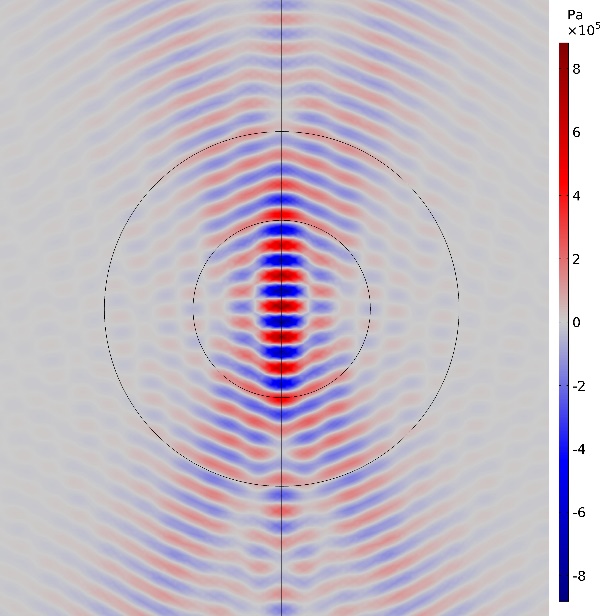 |
| --- | --- |
| **(b)** | **(a)** |
| **Fig S2. (a) Acoustic pressure (b) acoustic intensity distribution** | |

| 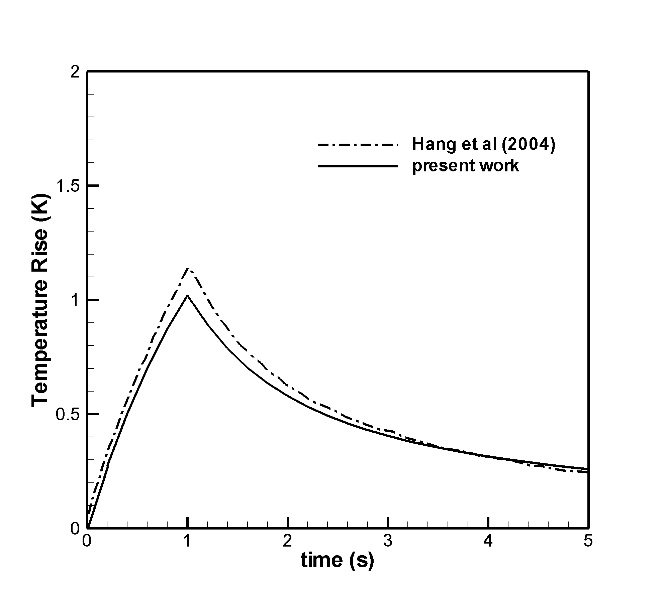 |
| --- |
| **Fig S3. Temperature rise at the focal point with time** |
